# Intersex Occurrence in Rainbow Trout (*Oncorhynchus mykiss*) Male Fry Chronically Exposed to Ethynylestradiol

**DOI:** 10.1371/journal.pone.0098531

**Published:** 2014-07-17

**Authors:** Sophie Depiereux, Mélanie Liagre, Lorraine Danis, Bertrand De Meulder, Eric Depiereux, Helmut Segner, Patrick Kestemont

**Affiliations:** 1 Unit of Research in Environmental and Evolutionary Biology (URBE-NARILIS), Laboratory of Ecophysiology and Ecotoxicology, University of Namur, Namur, Belgium; 2 Unit of Research in Molecular Biology (URBM-NARILIS), University of Namur, Namur, Belgium; 3 Centre for Fish and Wildlife Health, Vetsuisse Faculty, University of Bern, Bern, Switzerland; Institut National de la Recherche Agronomique (INRA), France

## Abstract

This study aimed to investigate the male-to-female morphological and physiological transdifferentiation process in rainbow trout (*Oncorhynchus mykiss*) exposed to exogenous estrogens. The first objective was to elucidate whether trout develop intersex gonads under exposure to low levels of estrogen. To this end, the gonads of an all-male population of fry exposed chronically (from 60 to 136 days post fertilization – dpf) to several doses (from environmentally relevant 0.01 µg/L to supra-environmental levels: 0.1, 1 and 10 µg/L) of the potent synthetic estrogen ethynylestradiol (EE2) were examined histologically. The morphological evaluations were underpinned by the analysis of gonad steroid (testosterone, estradiol and 11-ketotestosterone) levels and of brain and gonad gene expression, including estrogen-responsive genes and genes involved in sex differentiation in (gonads: *cyp19a1a*, *ER isoforms*, *vtg*, *dmrt1*, *sox9a2; sdY; cyp11b;* brain: *cyp19a1b*, *ER isoforms*). Intersex gonads were observed from the first concentration used (0.01 µg EE2/L) and sexual inversion could be detected from 0.1 µg EE2/L. This was accompanied by a linear decrease in 11-KT levels, whereas no effect on E2 and T levels was observed. Q-PCR results from the gonads showed downregulation of testicular markers (*dmrt1*, *sox9a2; sdY; cyp11b*) with increasing EE2 exposure concentrations, and upregulation of the female *vtg* gene. No evidence was found for a direct involvement of aromatase in the sex conversion process. The results from this study provide evidence that gonads of male trout respond to estrogen exposure by intersex formation and, with increasing concentration, by morphological and physiological conversion to phenotypic ovaries. However, supra-environmental estrogen concentrations are needed to induce these changes.

## Introduction

Gonads are unique among all organs given their bipotential nature: they can develop either into an ovary or a testis from a single primordium. The germinal epithelium is composed of germ cells which develop into sperm or eggs and somatic cells, involved in gamete maturation, which differentiate into Sertoli or follicular cells, depending on the destiny of the gonad. This leads to a plasticity of this developmental pathway. In mammals, the way by which cells choose between either fate is genetically controlled by the expression of a main male gene SRY that leads irrefutably to testis development [1]. Though these pathways are still not well understood in mammals, the situation is even more complex in fish, since they display a great variety of sex determination systems, and because sex differentiation can be subject to exogenous influences, which can override the presumptive developmental route (*i.e.* temperature in zebrafish) [2].

Sex steroids play a key role in these differentiation processes, acting as inducers of the developmental pathway and organizers of cellular differentiation [3]. Numerous studies in different fish species have demonstrated that the administration of exogenous hormones during the critical period of gonadal development can override endogenous sex determining/differentiating mechanisms in the developing fish, enabling the induction of phenotypic sex reversal. Given that one mating type can be more marketable, this practice is now commonly used in fish farming to obtain unisexual populations [4]–[6].

From an environmental perspective, this lability in gonad sexual differentiation leads to a sensitivity of fish to endocrine disruptors, which are molecules that mimic natural endogenous hormones or interfere with the endogenous synthesis of hormones. These molecules can reach surface waters through wastewaters if they are not properly removed by sewage treatment plants, and can locally attain harmful concentrations for wild fish [7]–[10]. The effective concentrations appear to be very low, being comprised within the ng to µg/L range, depending on the potency of the molecule and the duration of the exposure [10]. Nowadays there is considerable evidence that fish exposed to these chemicals display reproductive failures [11]–[13]. Among the endocrine disruptors, xenoestrogens such as the synthetic estrogen ethynylestradiol (EE2 - the main component of the contraceptive pill) in particular have attracted attention [14]. EE2 is widespread in surface waters with mean reported concentrations around 0.05 ng EE2/L but can attain higher levels locally, in exceptional cases up to 831 ng EE2/L [15]. Xenoestrogens can induce feminization of male fish, characterized by changes such as the production of the female egg yolk protein, vitellogenin (VTG, encoded by the gene *vtg*) by males and the appearance of ovarian-like morphological features within testes. Vitellogenin induction is recognised as a biomarker of xenoestrogenic exposure [16] and both immunochemical methods such as ELISAs for vitellogenin protein and RT-PCR methods for vitellogenin mRNA have been developed to measure it in a variety of fish species, including the rainbow trout (*Oncorhynchus mykiss*) [17]–[19]. Male fish displaying ovotestis (*i.e.* oocytes scattered within testis tissue) have been found in numerous fish species exposed to xenoestrogens in the field or in laboratory experiments [7], [20]–[23].

The rainbow trout is one of the most widely used fish species in ecotoxicology and constitutes a model organism in reproductive physiology of fish. It develops as a synchronous differentiated gonochorist teleost with an annual reproductive cycle [24], [25]. Sex determination is genetically controlled by a male heterogamety system (XX – XY) with a main male gene *sdY* recently found by Yano *et al.* (2012) [26]. Several studies have shown an impact of xenoestrogens and effluents on reproductive parameters in juvenile and mature rainbow trout [27], [28]. The labile period, during which gonad phenotypic differentiation is sensitive to steroid hormones, has been determined to be between 44 and 51 days post fertilization (dpf) [29]. Vitellogenin induction in trout in response to xenoestrogens is very sensitive [27], [30], with positive responses recorded at EE2 concentrations as low as 0.1 to 0.5 ng/L [31]. It has also been recorded in juvenile fish exposed to low concentrations of EE2 (1, 10 and 100 ng/L) [28]. While low concentrations of xenoestrogens are able to induce vitellogenin in trout, the situation is less clear with respect to ovotestis or intersex, as all studies on salmonids have failed to record morphological disturbances following exposure to xenoestrogens or effluents [27], [32]–[37]. Moreover, in their whole-lake experiment, Palace *et al.* [38] exposed fish to 5 ng/L of EE2 (nominal concentration), which induced elevated levels of VTG in Lake trout (*Salvelinus namaycush*), but remained without effect on gonad morphology, whereas fathead minnow (*Pimephales promelas*) and pearl dace (*Margariscus margarita*) also present in the same lake developed intersex gonads. Based on these considerations and to our knowledge, no study exists establishing intersex development in rainbow trout in response to xenoestrogenic exposure. This, however, does not mean that trout are not responsive at all. Indeed, high doses of estrogens, when used for aquaculture practices, are able to induce functional sex reversal of juvenile male rainbow trout into phenotypic females [39], [40].

The goal of the present study was thus to investigate the consequences of chronic exposure on gonad morphology of juvenile rainbow trout to EE2. Considering the apparently low propensity of the rainbow trout to develop ovotestis under chronic exposure to estrogen in environmental conditions (see above), we covered a large range of EE2 concentrations, from low, environmentally relevant (0.01 µg/L), to supra-environmental levels (0.1, 1 and 10 µg/L). The concentrations we used approached on the one side environmental levels (0.01 ug/L), although this concentration is still 10 to 20 times higher than what is usually measured in the environment (except in highly polluted sites, downstream of some sewage treatment plants), but it is 20 000 times lower than those used in aquaculture to reverse fish (*i.e.* 20 mg/kg of food [40] or 250 µg/L by immersion [39]). Special attention was devoted to the question as to whether xenoestrogen exposure would result in the formation of intersex gonads. To gain insight into the physiological changes associated with EE2 treatment, we also assayed gonad sex steroid levels (T, E2 and 11-KT) and expression of gonad and brain genes that are potentially regulated by xenoestrogen exposure. Another goal of our experiment was to characterize the whole transcriptome of the fish through the use of microarray assays. The results will be presented in another report. A first step was to measure the expression of key genes involved in sex differentiation and/or EE2 exposure by Q-PCR. The results of these analyses are presented here. As they are the first site of biological actions of environmental estrogens, the expression of the four isoforms of estrogen receptor (*ER*) genes was measured. The rainbow trout displays four nuclear ER isoforms, ERα1, ERα2, ERβ1 and ERβ2 [41] encoded by *esr1a, esr1b, esr2a* and *esr2b* genes, respectively. Attention has also been paid to genes involved in early sexual differentiation in rainbow trout [26], [42], [43]: *sdY* (sexually dimorphic on the Y chromosome), *dmrt1* (Doublesex and mab-3 related transcription factor 1) and *sox9a2* (sex determining region Y-box9alpha2). We also focus on key genes of the steroidogenesis, with *cyp11b2.1* (cytochrome P450, family 11, subfamily b, polypeptide 2.1), the gene encoding the enzyme 11β-hydroxylase involved in the 11-oxygenated androgen production; and the gonads and brain isoforms of the aromatase (*cyp19a1a, b*), the enzyme which synthesizes estradiol. Finally the well-established estrogenic exposure marker vitellogenin gene (*vtg*) was assayed.

## Materials and Methods

### 2.1. Ethics Statement

In this experiment, all animals were handled in strict accordance with the European Union’s guiding principles in the Care and Use of Animals (Directive 2010/63/EU) and the protocol was approved by the Animal Ethics Committee of the University of Namur (Permit Number: 10/149).

### 2.2. Fish and sampling

Three thousand eggs of an all-male rainbow trout (*Oncorhynchus mykiss*) population were imported at the eyed stage [23 days post fertilization (dpf)] from the experimental fish farm of the National Institute of Agronomic Research (INRA) (PEIMA, Sizun, France). The embryos were incubated until hatching in the bottom of nests at a constant temperature of 10°C (pH 8, O_2_: 9–10 mg/L, photoperiod LD 12∶12). These nests consisted of rectangular empty cubes (15×12×15 cm) surrounded by a fine thread hung on the flanges of 110-L closed circulating tanks. Fifty four percents of the fish reached hatching, which is close to values observed in experimental conditions [44]. After yolk sac resorption (60 dpf), the fry were randomly distributed into the tanks (108 individuals per tank) at an average water temperature of 13°C. At the onset of first feeding [Day 0 = D0 at 60 dpf], the fish were submitted to one of 5 nominal concentrations of 17α-ethynylestradiol (purity ≥98%, Sigma-Aldrich, Germany) solubilized in ethanol: 0 (solvent control), 0.01 µg/L, 0.1 µg/L, 1 µg/L and 10 µg/L, with 3 tanks per condition. Maximum ethanol levels were 0.002%. To reduce EE2 loss (due to adsorption, metabolic and microbial breakdown), 80% of the water was removed and replaced every 3 days (including new addition of ethynylestradiol). Actual EE2 concentrations were measured in each tank at 6 time points using the Quantitative Ethynylestradiol Enzyme Immunoassay (EIA) Kit (Marloie, Belgium) according to the manufacturer’s instructions. The fish were exposed chronically to EE2 for 76 days. At the end of the exposure, the fish weight and length were recorded, and the gonads and brain were collected from all fish. They were immediately frozen in liquid nitrogen and stored at −80°C until RNA extraction. The gonads from five to ten fish, as well as the brains from three fish per tank were pooled to reach enough material for further analyses. Moreover, the gonad pairs of six fish per tank were fixed in Bouin’s solution for histological analysis. It is worth noting that most of gonad samples were kept for microarray analysis, whose results will be presented in further reports.

### 2.3. Histological analysis

The gonads were removed from Bouin’s solution, dehydrated through a graded series of methanol, cleared with toluene, and embedded in paraffin. Sections (5 µm) were mounted on glass slides and stained with hematoxylin – eosin – safran trichrome (HES). To make sure ovotestis can be detected, transversal sections were taken over the entire gonad using at least 6 section planes. Each slide (minimum 6 per fish) was seen in its entirety to detect any signs of ovotestis. In total, 81 fish were analysed. Some slides were also stained with Masson’s trichrome (hemalun, phloxin and light green) to visualize the presence of cortical alveoli. The staging of the germ cells was done using the criteria of Billard 1992 [45] for trout male and Grier 2007 [46] for trout female. Female control samples were obtained from another experiment (Segner *et al.*, unpublished data). The fish were reared in clean water at a temperature of 7.5°C up to 180 dpf. Sampled fish were anaesthetized with MS 222 (buffered 3-aminobenzoic acid ethyl ester methanesulphonate, Argent Chemical Laboratories, Redmont, CA, USA). For histological analyses, fish were fixed in 4% buffered formalin. Samples were embedded in paraffin, 5 µm thick sections were prepared and stained by hematoxylin-eosin (HE).

Cautions were taken to avoid cross-contamination of samples during tissue collection or processing (*e.g.* cleaning of equipment between samples, use of different fixative containers, new solvants for dehydration,…) and the male fish were never in contact with female samples.

#### 2.3.1. Terminology

There exists a variable use of terms to describe intersexuality observed in fish in the literature. Here, we adapted the terminology proposed by Hecker *et al.* (2006) [47], with the term “ovotestis” describing the presence of oocytes into either normal or degenerated testicular tissue. The term “intersex” is the broader term refering to the occurrence of either female gametes in a male gonad or male gametes in a female gonad, while ovotestis explictely designates presence of oocytes into male gonads.

### 2.4. Sex steroids

The gonad concentrations of testosterone (T), Estradiol-17β (E2), and 11-keto-testosterone (11KT) were assayed by radio immunoassay (RIA), according to the protocol of Fostier and Jalabert (1986) [48]. Two pools containing 10 pairs of gonads per tank were assayed (n = 6 per treatment) separately. The steroids were extracted from gonads, following a procedure adapted from D’Cotta *et al.* (2001) [49]. Briefly, 1 g of frozen gonads were homogenized in 1 ml ethanol:H_2_O solution (50∶50). Homogenates were then centrifuged 15 min at 4000 g at 10°C, and supernatants were collected. The pellets were re-extracted in ethanol:H_2_O solution (80∶20). The supernatants were then extracted 3 times with dichloromethane, and the organic phase was conserved. These phases were evaporated to dryness and pellets were suspended in 200 µl ethanol. The extraction efficiency was measured with several testis samples spiked with 600 ng of E2. The mean efficiency (± SD) measured was 86.6±7%. All samples as well as the standards of E2, T and 11-KT (Sigma-Aldricht, USA) were assayed in duplicate. The anti-E2 and anti-T were supplied by the Hormonology Laboratory of Marloie (Belgium) and anti-11-KT was a gift from Dr. A. Fostier (INRA, Rennes, France); the radioactive hormones were purchased from Amersham Pharmacia (UK). No data are shown for the 1 µg/L group because of a technical accident which led to high mortalities and impede the sampling due to a lack of biological material.

### 2.5. Semi-quantitative Real-time PCR (SQ-RT-PCR)

#### 2.5.1. Total RNA extraction and RT

Total RNA from the gonads (2 pools per tank, containing 5 pairs of gonads each; in total n = 6 per concentration tested) and brain (3 pools per tank containing 3 brains each; in total n = 9 per concentration tested) were extracted using TRIzol reagent (Invitrogen, Life Technologies Europe B.V., Ghent, Belgium) as described previously [50]. Following extraction, samples were treated with DNAse (DNA-free kit, Ambion, Austin,USA) to avoid DNA contamination, according to the manufacturer’s instructions. The total RNA concentration was determined using an ISOGEN NanoDrop 2000c spectrophotometer (Wilmington, Delaware, USA) and RNA quality was further controlled on a Bioanalyzer 2100 (Agilent). Only samples with a RIN (RNA Integrity Number) >8 were kept for further analysis. To obtain cDNA, 4 µg of total mRNA was reverse-transcribed using the RevertAid™ H Minus First Strand cDNA Synthesis Kit (Fermentas, Germany) according to the manufacturer’s instructions. Negative reverse transcriptions controls were performed by omitting reverse transcriptase from the RT step in order to validate CT values and check we avoid genomic DNA amplification.

#### 2.5.2. Primer design

Key genes involved in sex differentiation in gonads (the four *ER isoforms*, *dmrt1, sox9a2, sdY, cyp11b2.1, cyp19a1a, vtg*) and the brain (the four *ER isoforms* and *cyp19a1b*) were selected in order to investigate their expression under the different experimental conditions. Specific primers were selected from the literature or were newly designed using Primer3 software [51] (Table 1). The newly-designed primers respected, whenever possible, the following restriction parameters: length, 21–23 base pairs (bp); no more than four successive identical nucleotides; guanin-cytosin content, 30–70%; a maximum of two guanines or cytosines among the five 3′-end bases; no primer dimers; and a short amplicon size (70–150 bp). Moreover, each pair was chosen with at least one primer flanking an intron-exon boundary to prevent genomic amplification. The localization of introns/exons boundaries was made from Leroux *et al.* (1993) [52] annotation of the ER gene. To further assessed their identity, each primer was then blasted using BLASTN alignement tool [53] against each ER isoforms as well as the entire rainbow trout nucleotides collection. All primers were purchased from Eurogentec (Seraing, Belgium).

**Table 1:** Primer sequences used for Q-PCR analysis.

Gene		Sequence	Reference sequence	Efficiency	Reference
***esr1a***	F	ACA-GGA-ATC-GTA-GGA-AGA-GCT-G	AJ242741	2.05	Newly designed
	R	CGT-AGG-GTT-TCT-CTC-TGT-CAC-C	AJ242741		
***esr1b***	F	ACC-AGG-GTG-AAA-GCT-GTC-TGC-TA	DQ177438	1.98	Newly designed
	R	CTA-AGA-GGG-ATA-GAG-GAA-AGG-AGA	DQ177438		
***esr2a***	F	AGA-CGG-TCA-TCT-CGC-TGG-AAG	DQ177439	1.95	Newly designed
	R	ACA-CTT-TGT-CAT-GCC-CAC-TTC-GTA	DQ177439		
***esr2b***	F	AGA-GGA-AGT-GAA-CTC-CTC-CTC-AGG	DQ248229	2.02	Newly designed
	R	GAT-AGT-AGC-ACT-GGT-TAG-TTG-CTG-GAC	DQ248229		
***dmrt1***	F	GGA-CAC-CTC-CTA-CTA-CAA-CTT-CA	AF209095	2.03	[77]
	R	GTT-CGG-CAT-CTG-GTA-TTG-TTG-GT	AF209095		
***sox9a2***	F	ATG-CAG-GTG-CCC-AAG-GCT-CA	AB006448	2.09	[43]
	R	CTC-TGG-CTG-GGG-CTC-ATA-TA	AB006448		
***sdY***	F	GTG-GTT-TTA-AGC-TCT-AGG-GAG-GA	AB626896	1.97	[26]
	R	GAG-TGA-TGA-GTC-TTG-TCC-AAA-C	AB626896		
***cyp11b2.1***	F	CTG-GGA-CAT-GTG-TCC-AGG-CG	AF179894	1.98	[96]
	R	CTG-GAT-CCT-GAA-ACA-CAT-CT	AF179894		
***Vtg***	F	ACC-CTG-AAC-CGG-TCT-GAA-G	BX084166	2.06	Le Gac et al.(personal communication)
	R	CAG-TAT-CTG-CTC-CAC-CAC-A	BX084166		
***hprt1***	F	AAG-CAG-CCC-CTG-TGT-TGT-GA	TC55247	2.0	Leder *et al.*(personal communication)
	R	CGG-TTT-AGG-GCC-TTG-ATG-TA	TC55247		
***cyp19a1a***	F	CTC-TCC-TCT-CAT-ACC-TCA-GGT-T	BX083177	1.98	[43]
	R	AGA-GGA-ACT-GCT-GAG-TAT-GAA-T	BX083177		
***cyp19a1b***	F	CTG-GCA-AAC-GGT-TCT-GAT-C	AJ311937	2.1	[101]
	R	TGA-TGG-ACA-GAG-TGT-CTG-G	AJ311937		

#### 2.5.3. Quantitative Real-Time PCR

Real-time PCR was performed in 20 µl (5 µl of cDNA, 2.5 µl of each primer at 500 nM, 10 µl MasterMix 2x) with SYBR Green (Applied Biosystems, Foster City, California, USA) as an intercalating agent. Each measurement was performed in duplicate. The PCR conditions were: 10 min at 95°C, 40 cycles: 15 sec at 95°C, 1 min at 60°C. The specificity and identity of the RT-PCR products were checked by performing a dissociation curve (gradient from 60°C to 95°C) for each gene.

Relative quantifications were established by the comparative CT method (also known as the 2^−ΔΔCt^ method) [54]. This method assumed that the PCR efficiencies of target and reference genes are approximately equal [55], which was validated in our experiment. PCR efficiencies were calculated from high linearity standard dilution curves (Pearson correlation coefficient R^2^>0.985) slopes according to the equation E = 10^(−1/sl^°^pe)^ (Table 1). Relative gene expressions are represented as the fold change in gene expression normalized to an endogenous reference gene (housekeeping gene) and relative to the untreated control (0 µg/L EE2), following these equations: ΔΔCt = (C_T,Target_ – C_T,Housekeeping_)_Test_ – (C_T,Target_ – C_T,Housekeeping_)_Control_, and Fold change = 2^−ΔΔCt^. *hprt1* (the hypoxanthine phosphoribosyltransferase 1, a nucleoside metabolism enzyme) was chosen as the housekeeping gene in our experiment. It was selected among several candidate housekeeping genes mentioned in the literature as relevant for gene expression measurement after EE2 exposure, after effective validation of its expression in our samples [42], [56].

### 2.6. Statistical analysis

The results were analyzed by one-way analysis of variance (ANOVA) with linear regression and lack of fit to linearity with EE2 concentration as the fixed criterion and independent variable [57] using the Excel software. Homoscedasticity was checked by the Bartlett test. When heteroscedasticity was significant (p<0.05), data were log-transformed. When the lack of fit to linear regression was significant (p<0.05), the ANOVA1 was followed by Sheffe’s post-hoc comparisons [57] (p<0.05 threshold), to point out significant and physiologically relevant variations between conditions. All data are presented as the mean of the replicates and the error bars represent ± 2 standard errors of the mean (SEM), i.e. an approximation of the 95% confidence interval of the mean of each group. A Fisher’s exact test was performed to analyse if any differences in frequencies in gonad morphologies observed in our study occured between the triplicate tanks.

## Results

The mean EE2 concentrations ± SD were 0.08±0.06 µg/L; 1.62±1.74 µg/L and 9.88±5.06 µg/L. The 0.01 µg/L EE2 concentration was under the detection limit (set at 0.02 µg/L).

The exposure of trout fry to ethynylestradiol impaired their growth rate at the high concentrations used, with a significant drop in the size and weight of the fish exposed to 1 and 10 µg/L compared to the controls (p<0.05). Fish size, weight and mortality observed following the 76 days of exposure are given in Table 2. No effect of fish densities were observed on fish size and weight.

**Table 2:** Fish size, weight and mortality.

EE2 concentration (µg/L)	Number of fish	Mean size (cm) ± SD	Mean weight (g) ± SD	Mean mortality (%) ± SD
0	289	6.3±1.49	8.1±0.65	10.8±6
0.01	283	6.3±1.32	8.01±0.57	12.7±6
0.1	270	6.05±0.3	7.8±0.66	16.7±5
1	101	2.93±0.7*	5.75±0.5*	68.8±9
10	208	2.58±0.4*	5.34±0.34*	35.8±3

*significantly different from control (p<0.05).

Fish size, weight and mortality observed following 76 days of exposure (from 60 to 136 dpf) to several doses of ethynylestradiol (EE2). Mean values ± SD for 3 replicates tanks are provided.

### 3.1. Histology

Alterations of gonad morphology were observed for all EE2 concentrations tested. In general, histological analysis of gonad sections identified common features due to EE2 treatment, but also revealed features specific to each EE2 concentration. Three categories of fish gonad morphology could be distinguished, namely testicular, ovotestis, and ovarian-like. A description of each of these categories is given below. The proportion of fish belonging to each of these categories per experimental condition is presented thereafter.

#### 3.1.1. Testicular morphology

**Figure 1:**
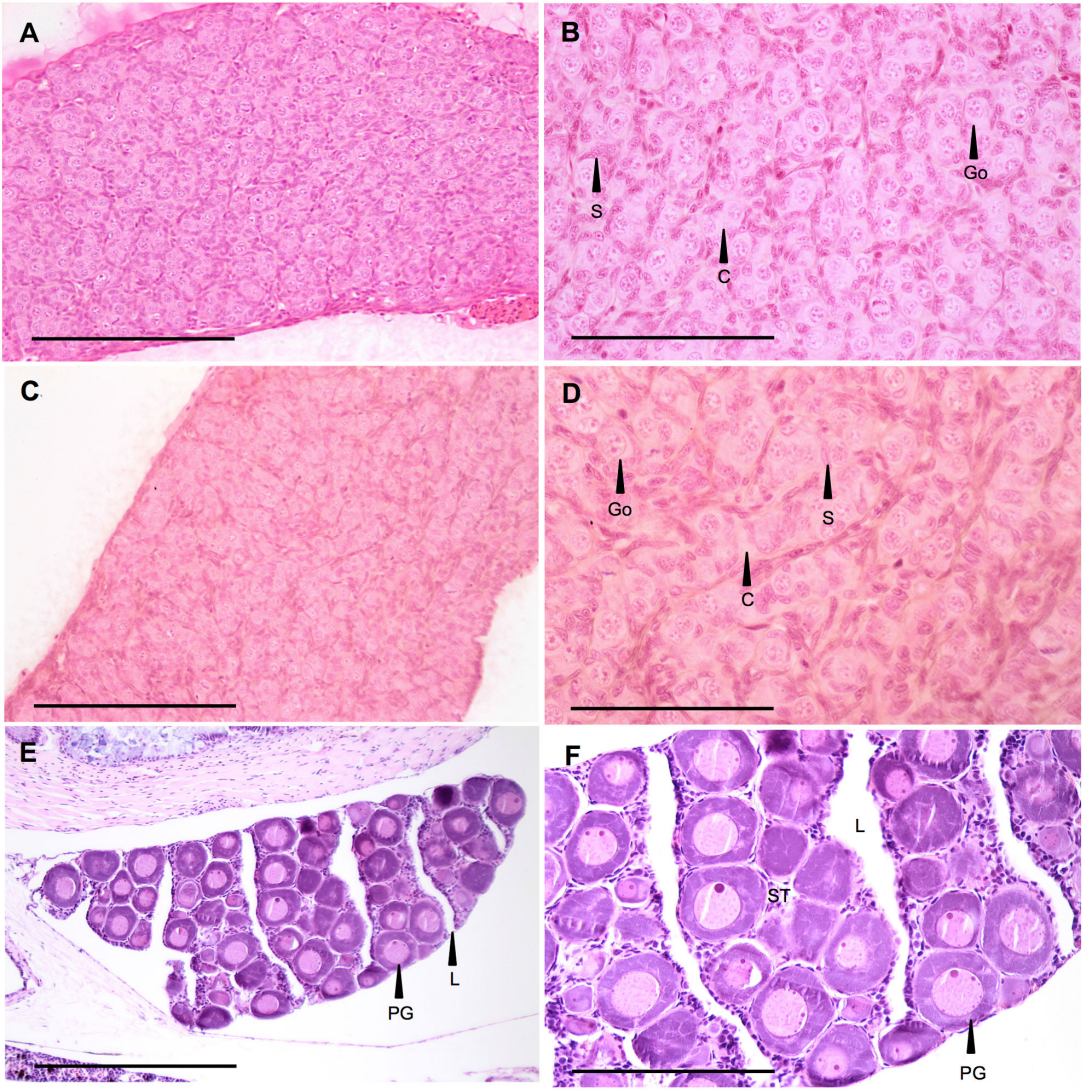
Testicular morphology. A–B: Rainbow trout testis at 136 days post fertilization (dpf), control fish (1360 degree-days). This picture shows the differentiation status of a control testis, with a dominance of gonocytes (Go, future spermatogonia) surrounded by supporting cell (S, future sertoli cell) organized in cords (C, future tubules). HES. Scale bar A = 100 µm, B = 50 µm. C–D: Rainbow trout testis at 136 dpf, fish exposed chronically to 0.01 µg/L of EE2 for 76 days. HES. Go: gonocyte; S: supporting cell. Scale bar C = 100 µm, D = 50 µm. E–F: **Normal ovarian morphology**. Rainbow trout early stage ovarian morphology, control fish (1350 degree-days). PG: primary growth oocyte; L: lamellae; ST: stroma. Scale bar E = 100 µm, F = 50 µm.

Macroscopically, the gonads of all-male rainbow trout at 136 dpf appeared as paired and tubular elongated structures lying along the dorsal side of the abdominal body cavity. Histologically, the gonads displayed the histological features of a differentiated yet immature testis, as described in the literature [25], [45], [58]. It was characterized by early seminiferous tubules or cords containing undifferentiated gonocytes (future spermatogonia) surrounded by several supporting cells (future Sertoli cells) in the interstitium between the tubules (Figure 1 A–D). This morphological feature was observed in all control fish (Figure 1 A–B), as well as in several fish exposed to EE2 at the lower concentration used (0.01 µg/L) (see section 3.1.5), covering the entirety (Figure 1 C–D) or areas of the gonad (see ovotestis morphology section 3.1.2). A normal ovarian morphology at the same developmental stage is given in Figure 1E–F.

**Figure 2:**
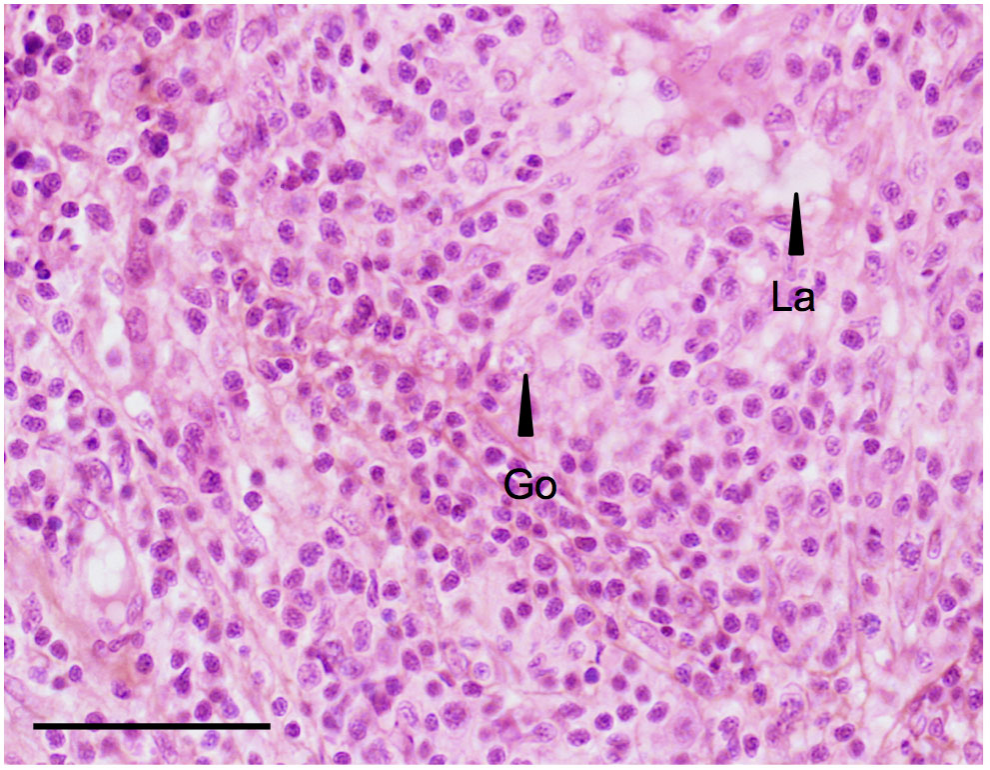
Altered testicular morphology. All-male rainbow trout testis at 136 dpf exposed chronically to 1 µg/L EE2. HES. Scale bar = 50 µm. This picture illustrates the degeneration of the testis observed in several fish exposed to EE2, such as a loss of tubular arrangement, loss of germ cell number (Go) and differentiation, presence of lacuna (La). There is no recognizable differentiation of the gonad into male or female phenotype.

**Figure 3:**
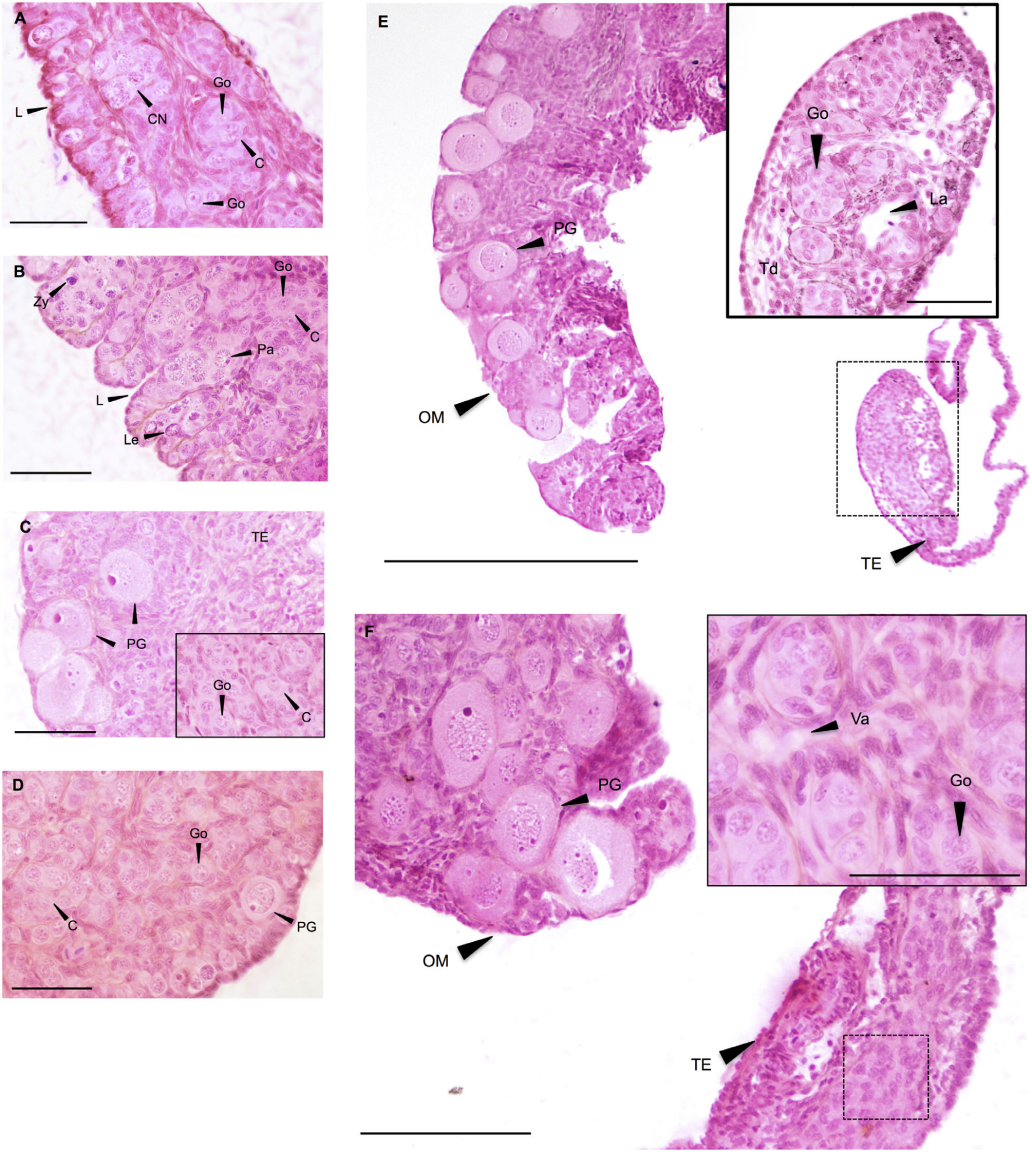
Intersex morphology. **A–B: Ovotestis 1**. All-male rainbow trout testis at 136 dpf exposed chronically to 0.01 µg/L and 0.1 µg/L for 76 days. HES. L: lamellae; C: cords of gonocytes (Go); CN: cell nest in different meiosis stages: Le: leptotene; Zy: zygotene; Pa: pachytene. Scale bar = 50 µm. **C–D:** **Ovotestis 2**. All-male rainbow trout testis at 136 days post fertilization (dpf) exposed chronically to 0.01 µg/L EE2 for 76 days. HES. Scale bar C = 50 µm, insert in C = 25 µm, D = 50 µm. Figure C shows oocytes appearance in an altered testis structure. The insert in Figure C shows the normal testis structure, with gonocytes (Go) organized in cords (C) observed in other areas of this gonad. Figure D shows oocyte appearance in a normal testis structure. PG: primary growth oocytes; TE: altered testicular tissue; FC: follicular cells. **E–F:** **Ovotestis 3**. All-male rainbow trout testis at 136 dpf exposed chronically to 0.01 µg/L for 76 days. HES. Inserts show the altered testis structure at higher magnification. Dotted line represents the enlarged tissue section. OM: ovarian-like morphology; PG: primary growth oocytes; TE: altered testicular tissue; Sg: spermatogonia, La: lacune; Td: tubule disorganization; Va: vacuolation. Scale bar E = 300 µm, F = 100 µm, inserts = 50 µm.

In treated fish, the testis could show different degrees of degeneration (i.e. loss of tubular arrangement, loss of germ cell number and differentiation, presence of lacuna) which could occupy small areas of the testis or could extend over the whole tissue (see Figure 2 and Figures 3 E–F). Based on these considerations, several individuals display gonads containing germ cells without any recognizable differentiation into male or female phenotype. Since our study was conducted on a all-male rainbow trout population, they were specified as having “altered testicular” morphology (Figure 2).

#### 3.1.2. Ovotestis morphology

Ovotestis gonads were observed only in the EE2 treatments. An ovotestis phenotype is characterized by the presence of oocytes within the testis. Depending on the number and maturation stage of the oocytes in the testis, three categories of ovotestis were distinguished:

*Ovotestis 1:* the normal and degenerated testis structures reminiscent of ovigerous lamellae, i.e. blisters containing groups of meiotically dividing oogonia. No large previtellogenic follicles are seen, but the germ cells display meiotic stages. The majority of the gonads display a normal testicular morphology, with most sperm cells (gonocytes) being at the spermatogonial stage in cord structure (Figure 3 A–B). With respect to the female germ cells, ovotestis 1 shows similarities to the morphology of an early ovarian developmental stage [46], [59], [60].
*Ovotestis 2:* presence of isolated oocytes within an otherwise normal and/or degenerated testis structure (Figure 3 C–D). The oocytes display the characteristics of primary oocytes (protoplasmic oocyte or previtellogenic follicles). From the diplotene phase onward, the oocytes are surrounded by early follicular cells [46]. The oocytes are dispersed among the gonocytes, albeit most of them appeared mainly in the peripheral part of the testis. The arrangement of oocytes in ovigerous lamellae, as is characteristic for ovaries, was not seen.
*Ovotestis 3:* gonads are separated into an ovarian part, where oocytes but no spermatognia can be found, and a testicular part. The latter shows a degenerative morphology (Figure 3 E–F). The oocytes are usually in the primary growth stage (Fig. 3 E–F). However, in some individuals, mature oocytes containing much vitellogenin are also observed (see section 3.1.4.2). For all samples belonging to this category, serial sections were made and confirmed that the asymetry was not restricted to one single area within the gonad, but was consistently present throughout the entire gonad.

The classification above is based exclusively on the criteria “number of oocytes” and “maturation stage/organization of oocytes”. The testicular alterations are neither described in details in this article nor are they considered in the ovotestis classification, since this was not within the scope of this study.

#### 3.1.3. Ovarian-like morphology

**Figure 4:**
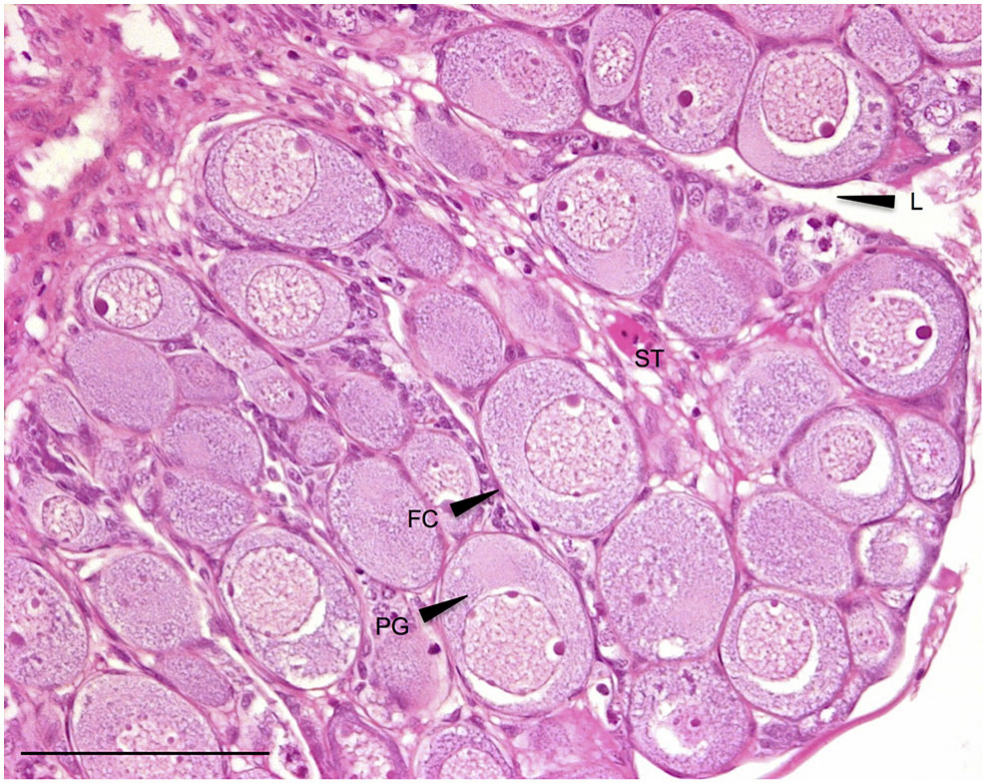
Ovarian-like morphology (sex-reversed fish). All-male rainbow trout testis at 136 dpf exposed chronically to 1 µg/L EE2 for 76 days. HES. FC: follicular cell; PG: primary growth oocyte; L: lamellae; ST: stroma. Scale bar = 100 µm.

Several fish subjected to the EE2 treatments displayed ovary-like gonads, i.e. they were apparently gender-converted (at least in terms of structure) (Figures 4). The gonads displayed the characteristics of an immature ovary, with an ovarian germinal epithelium forming ovigerous lamellae inside the ovarian cavity [25], [59], [61], [62]. The mesenchymal stroma underlying the multi-layered epithelium was composed of fibroblasts, interstitial cells, leukocytes, blood capillaries and collagen fibers [25],[63],[46]. At least in some individuals, it appeared that the area of the stromal tissue (ST in figure 4) between the follicles was increased compared with that expected in a “normal” ovarian morphology of genetic females at this developmental stage.

#### 3.1.4. Treatment-associated morphological particularities of the gonads

**Figure 5:**
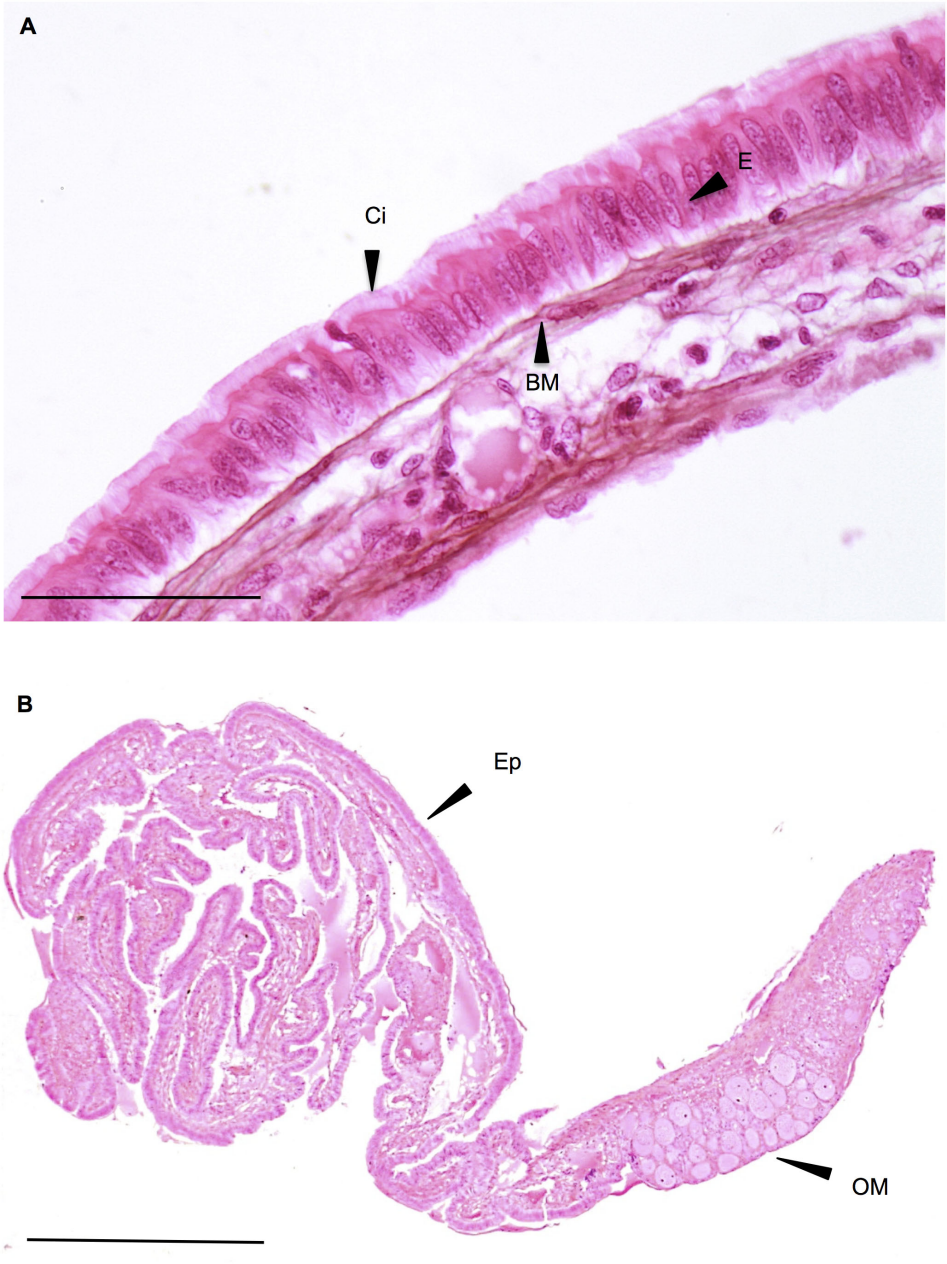
Proliferative oviduct-like epithelioid structure. All-male rainbow trout testis at 136 dpf exposed chronically to 1 and 10 µg/L EE2 for 76 days. HES. Ci: cils; E: columnar epithelial cells; BM: basement membrane; Ep: epithelioid; OM: ovarian-like morphology. Scale bars A = 50 µm; B = 500 µm.

*Oviduct-like epithelioid structure:* Some fish exposed chronically to EE2 displayed an epithelial structure extending from the gonad. The epitheloid was composed of ciliated columnar cells lying upon thin loose vascular connective tissue (Figure 5A). The structure contained many circumvolutions, was always connected to the gonad, and displayed blind ends. It could reach huge proportions, sometimes exceeding the size of the gonads (Figure 5B). Oocytes were never observed in this struture. This oviduct-like structure was exclusively observed at the higher EE2 concentrations (0.1, 1 and 10 µg/L). The percentages of fish displaying this feature per concentration were: 70% (14/20) at 0.1 µg/L; 78% (7/9) at 1 µg/L and 83% (15/18) at 10 µg/L. The size of the structure appeared to increase with the increase in EE2 concentration, attaining huge proportions at 10 µg/L, compared to the gonad size (Figure 5B).

**Figure 6:**
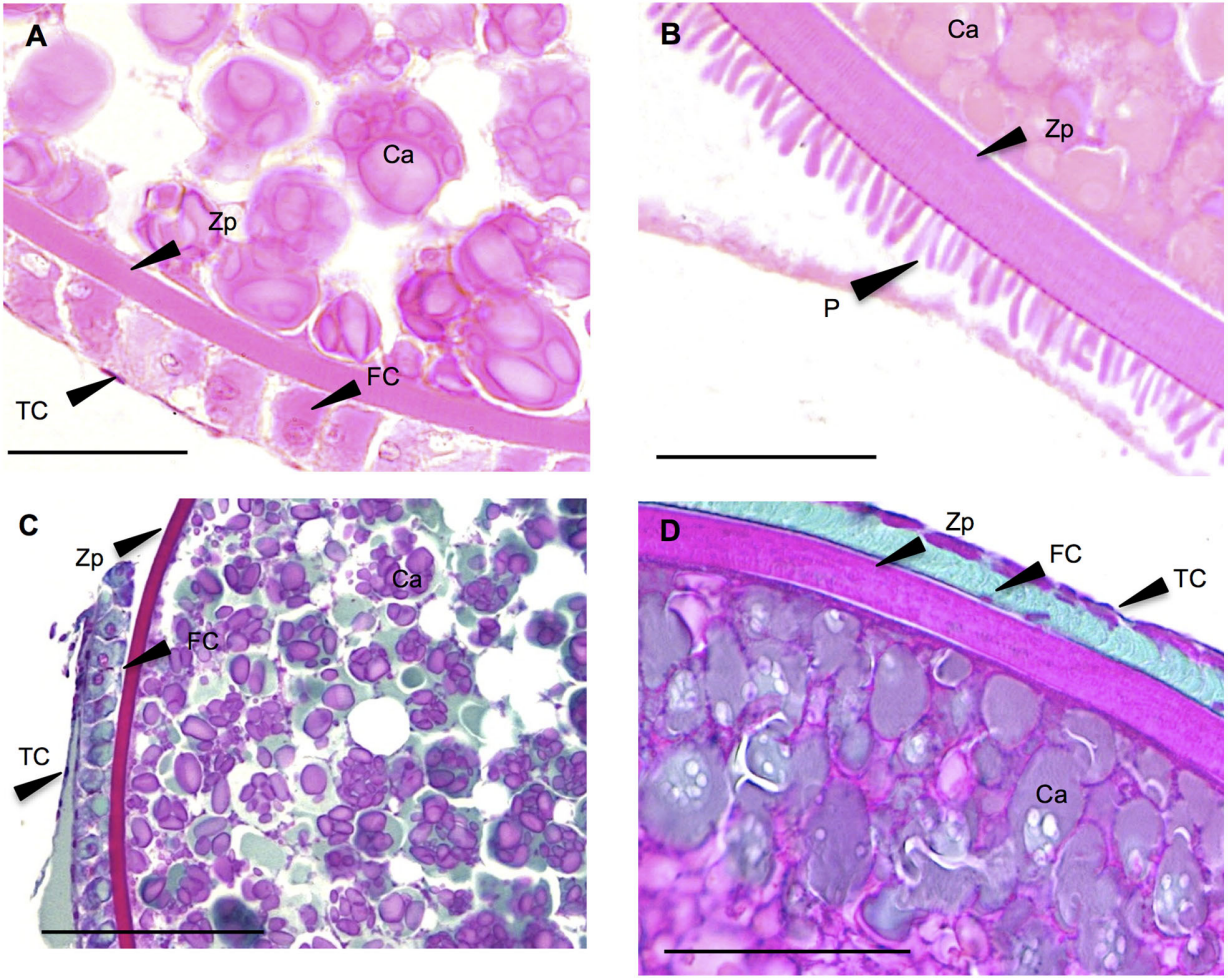
Vitellogenic follicles. **A–B**: Vitellogenic follicles found in gonads of all-male rainbow trout testis at 136 dpf exposed chronically to 0.01 and 10 µg/L EE2 for 76 days. HES. Zp: Zona pellucida; FC: follicular cells (granulosa); TC: thecal cells; CA: cortical alveoli; P: protrusions probably originated from the follicular cells. **C–D**: Masson’s staining of a vitellogenic follicle. Scale bars 50 µm.

*Vitellogenic oocytes:* Surprisingly, we found vitellogenic follicles in several juvenile fish exposed to EE2 (Figure 6 A–D). Their size was between 0.8 and 1.5 mm. They were filled with cortical alveoli and vitellogenic deposits uniformly distributed in the ooplasm (Figure 6 A,C), or aggregated on its periphery (Figure 6 B,D). Their walls displayed features of vitellogenic follicles, with the zona radiata surrounding the oocyte, covered by the granulosa layer and the thecal cells. However, the morphology of the folliclar layer deviated in two aspects from the normal morphology (Figure 6 A–B). In figure 6A, the granulosa is composed of a regular continuous cuboidal cell layer, as described in mature rainbow trout by Van den Hurk (1979) [62]; in figure 6B, however, the oocyte displays an acellular layer composed of microvilli-like structures representing the zona pellucida (chorion). These protrusions may originate from the follicular cells. The results of Masson’s trichrome staining supported the presence of aberrant follicular morphology (Figure 6 C–D), and the phloxine staining suggested the presence of proteins in the cortical alveoli. The vitellogenic follicles were floating in the body cavity, separated from the gonad. Intermediary stages between the previtellogenic oocytes (stage 2) and the vitellogenic follicles (stage 5) were not found. In total, five of all fish examined displayed mid-vitellogenic follicles, among all the concentrations tested. At 0.01 µg/L (two follicles observed), one of the follicles appeared immediately adjacent to testicular tissue. These results were absolutely unexpected, as the fish were juvenile and no other signs of maturation were found in the gonads displaying these follicles.

#### 3.1.5. Relative frequencies of the morphological gonad forms

**Figure 7:**
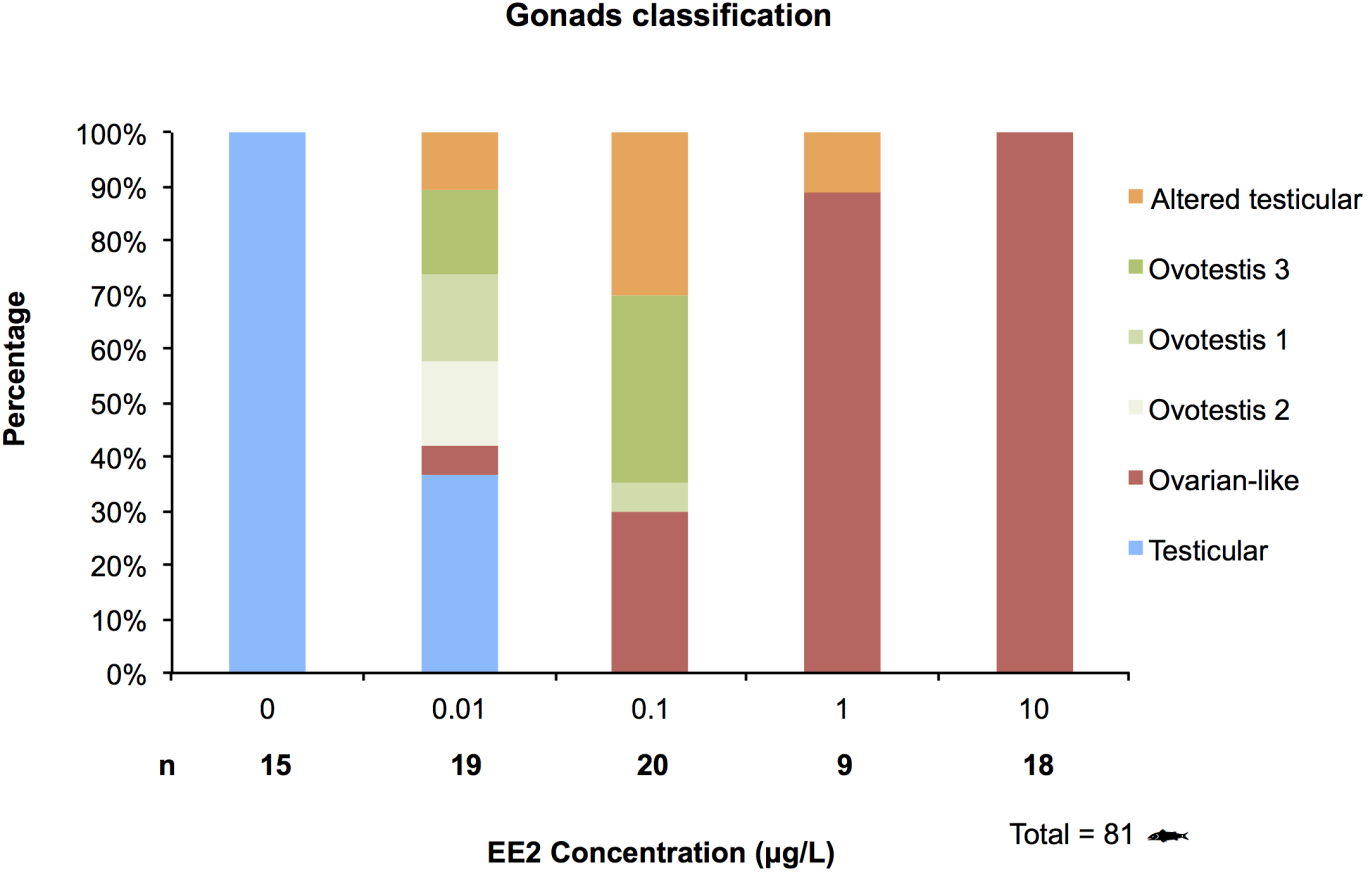
Relative frequencies of the gonad mophological forms. This graph represents the percentage of fish belonging to each morphological category, per concentration of EE2 used in the chronic experiment. n refers to the number of fish analyzed per experimental concentration.

Figure 7 presents the gonad morphology found at each percentage in the individual experimental treatments. No differences of frequencies in gonad morphologies were seen between replicate tanks (Fisher’s exact test: p = 0.85 in the 0.01 µg/L group; p = 0.94 in the 0.1 µg/L group).

All fish from the control tanks belonged to the testicular category, with male fish displaying immature testes. The presence of female features within the testis appeared in the groups exposed to EE2 at 0.01 µg/L and higher. In the groups exposed to the lowest EE2 concentration (0.01 µg/L), nearly half of the group (47.4%) displayed intersex gonads. The three ovotestis categories were equally represented, with 15.8% of the fish belonging to each category. Several individuals displaying an altered testicular morphology were also observed, as well as one case of an ovarian-like morphology.

At higher concentrations, an increasing percentage of fish showed ovary-like gonads. At 0.1 µg/L, one third of the fish were reversed (30%), while ovotestis individuals still represented about 40%, with ovotestis 1 being the main stage represented (35%). Only one individual displayed ovotestis 3 (asymetric). No fish with histological testes were seen at this concentration. All the fish exposed to 1 and 10 µg/L of EE2 appeared reversed, with only one individual displaying an altered testicular morphology at 1 µg/L.

### 3.2. Sex Steroids

**Figure 8:**
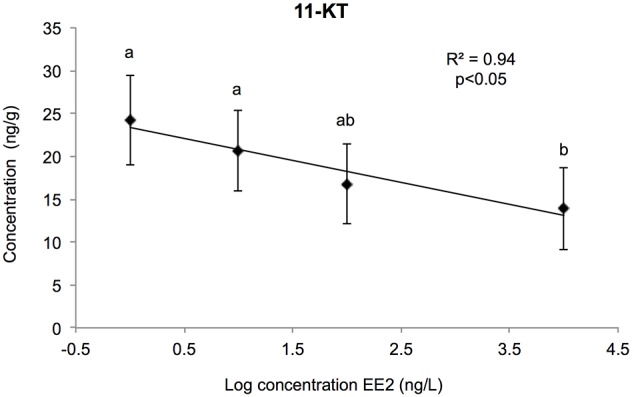
11-ketosterone levels. This graph show the linear relationship between [11-ketosterone] and LOG[EE2] in rainbow trout fry gonads submitted chronically to increasing concentrations of EE2. For each group, data represents the mean ± 2 SEM from 6 replicates measured independantly. Each replicate consisted of a pool of 10 pairs of gonads.

Testosterone (T), estradiol (E2) and 11-ketotestosterone (11KT) gonads levels were assayed to determine whether chronic EE2 exposure was able to disrupt the steroidogenesis in trout fry. At the concentrations tested, the levels of 11-ketotestosterone decreased linearly with the increase of the log of EE2 concentration (R^2^ = 0.94; p<0.05) (Figure 8). These levels ranged between 24.2±5.2 ng/g in the control group and 13.9±4.7 ng/g in gonads exposed to the highest EE2 concentration.

The levels of E2 and T (mean values of 6 replicates for all the concentrations tested ± 2 SEM) did not vary significantly between the concentrations of EE2 tested (4.76±0.51 and 13.4±1.7 ng/g, respectively).

### 3.3. Gene Expression

#### 3.3.1. Gonads

**Figure 9:**
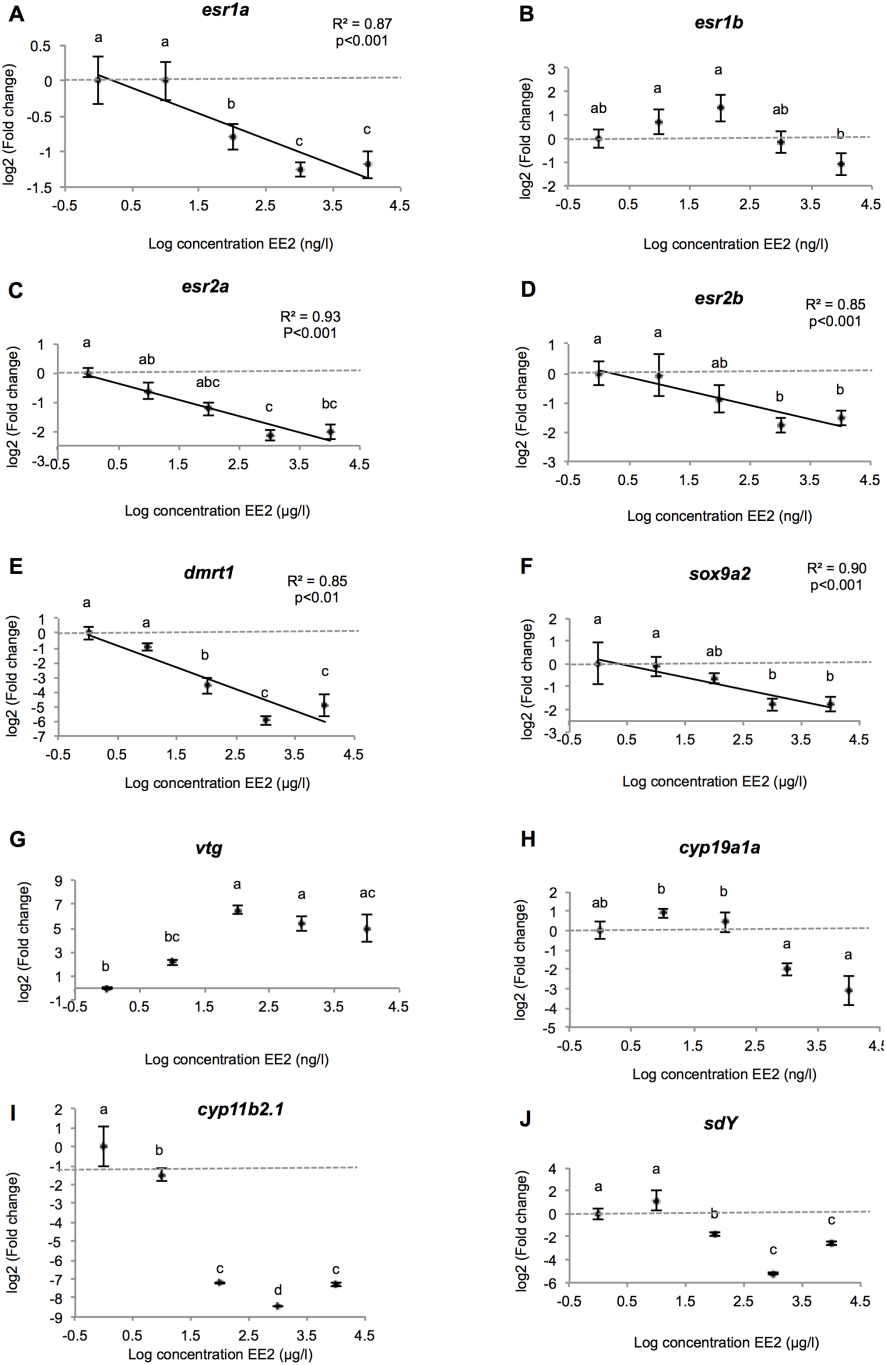
Genes expression profiles in the testis. Relationship between fold change (expressed as mRNA relative expression ratio with control group) of differentially expressed genes and LOG[EE2] in the testis of rainbow trout fry exposed chronically to increasing concentrations of EE2. For each group, data represents the mean ± 2 SEM from 6 replicates measured independantly. Each replicate consisted of a pool of 5 pairs of gonads. The letters a, b, c summarize the post hoc comparisons (p<0.05), the groups with the same letter being not significantly different. When the lack of fit to linear regression is not significant (p>0.05) the linear regression and associated R^2^ are shown.

We measured the expression of the four isoforms of estrogen receptor (*ER*) genes (*esr1a, esr1b, esr2a, esr2b)*, as well as four “male genes” (*sdy, dmrt1, cyp11b* and *sox9a2*) and two “female genes” (*cyp19a1a* and *vtg*). The expression of all genes measured varied significantly according to the treatment. Moreover, within the range of concentrations tested, most of the genes tested showed a positive or negative linear response to EE2 (Figure 9 A–J).

Three of the four ER isoforms decreased linearly with the increase in EE2 levels (p<0.05). They showed under expression of a similar amplitude, with a fold change between the control and 10 µg/L of EE2 of 5.9, 4.2, and 2.9-fold decrease in expression of *esr1a*, *esr2a* and *esr2b*, respectively (see expressions in log2 fold change in Figure 9 A, C, D). Only the *esr1b* isoform showed a different pattern of expression. It increased at the intermediate EE2 concentration with a 2.45 fold induction at 0.1 µg/L and then decreased significantly (p<0.01) at 10 µg/L, with its expression reduced by 50% compared with the control.

We observed a linear decrease in the expression of the “male genes” *dmrt1* (R^2^ = 0.85; p<0.001) and *sox9a2* (R^2^ = 0.93; p<0.001) (Figure 9 E–F). Sharp underexpression was seen for *dmrt1* which was 30 times less expressed at 10 µg/L than in the control group. The expression of *sox9a2* varied to a lesser extent, its expression being reduced to a fold change of 3.4 at 10 µg/L compared with the control. *sdY* and *cyp11b2.1* expressions were strongly repressed by the EE2 treatment, with up to 38 and 343-fold decrease at 1 µg/L respectively (Figure 9 G–H). Both genes displayed lesser underexpression at the 10 µg/L concentration.

Expression of the “female genes” varied, as shown in Figures 9 G–H. The *vtg* gene was overexpressed compared with the control group, with a significant peak (p<0.001) at 0.1 µg/L (fold change of 91.2). This overexpression was still significantly different from the control group but declined to a fold change of 39.9 and 30.9 at 1 and 10 µg/L, respectively. At 0.01 µg/L of EE2, the *vtg* overexpression reached a fold change of 4.41, which was not significantly different from the control group.

Gonad aromatase gene expression showed slight but non-significant overexpression at 0.01 and 0.1 µg/L, with fold changes of 1.8 and 1.3 compared with the control group, respectively. Its expression decreased significantly at the higher concentrations, being 4 and 9 times underexpressed at 1 and 10 µg/L, respectively.

#### 3.3.2. Brain

**Figure 10:**
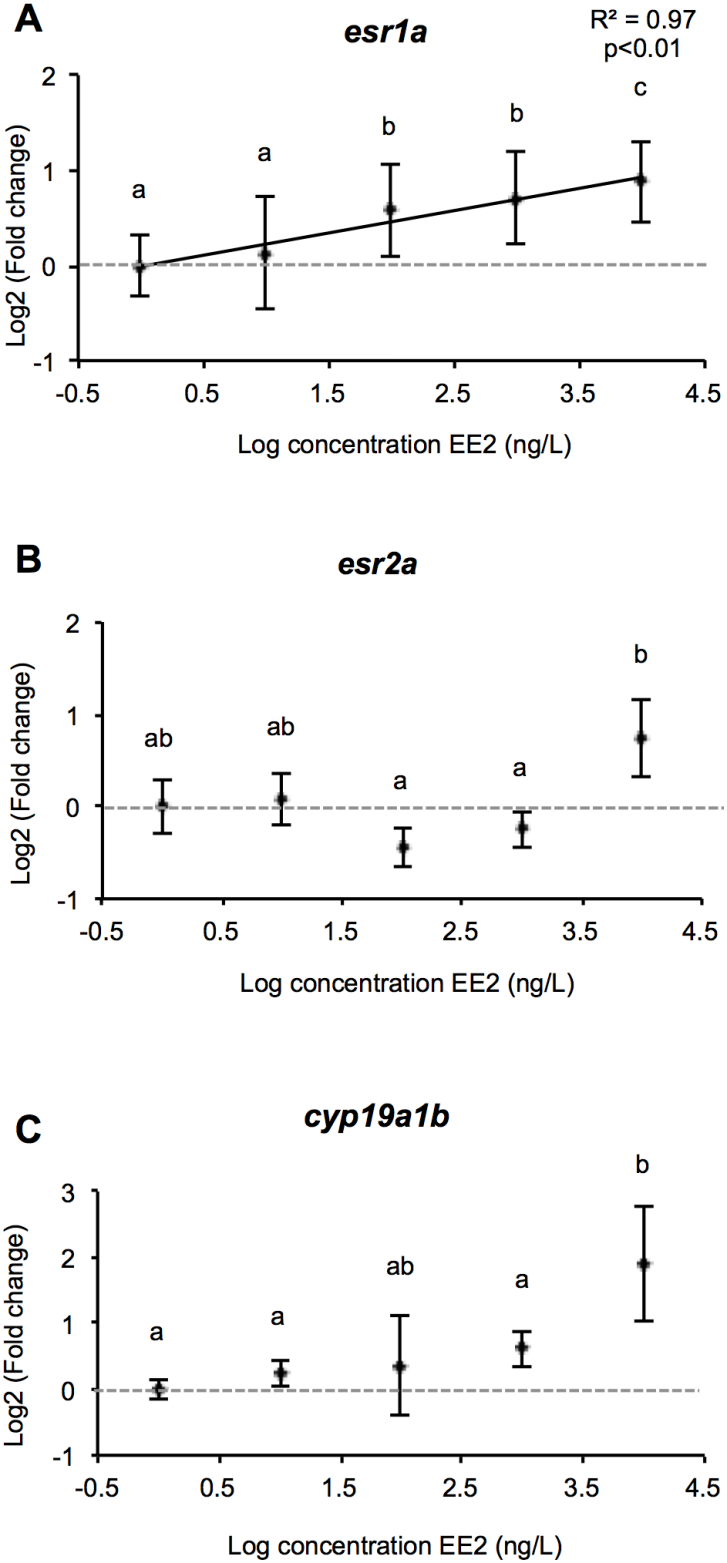
Genes expression profiles in the brain. Relationship between fold change (expressed as mRNA relative expression ratio with control group) of differentially expressed genes and LOG[EE2] in brains of juvenile male rainbow trout gonads exposed chronically to increasing concentrations of EE2. For each group, data represents the mean ± 2 SEM from 9 replicates measured independantly. Each replicate consisted of a pool of 3 brains. The letters a, b, c summarize the post hoc comparisons (p<0.05), the groups with the same letter being not significantly different. When the lack of fit to linear regression is not significant (p>0.05) the linear regression and associated R^2^ are shown.

We measured the expression of the four isoforms of *ER* genes, as well as the brain aromatase form, *cyp19a1b*. Only the *esr1a* isoform displayed a significant linear increase (R^2^ = 0.97; p<0.001) among the concentrations of EE2 tested, with an amplitude of overexpression of 1.8 between the control and the 10 µg/L groups (Figure 10 A). The variations observed in *esr2a* expression were less clear. There was no significant difference between the control group and all the concentrations tested. However, a small decrease in its expression at 0.1 and 1 µg/L of EE2 and an increase at 10 µg/L created a significant difference between these groups, the expression at 10 µg/L being higher by a factor of 0.8 (p<0.01) compared with 0.1 and 1 µg/L (Figure 10 B). The changes in *esr1b* and *esr2b* expression did not differ significantly between the EE2 treatments (data not shown).

The expression of *cyp19a1b* increased slightly with the rise in EE2 concentration, and reached a significant peak at 10 µg/L with a fold change of 3.7 observed against the control group (p<0.001) (Figure 10 C).

## Discussion

This study showed disruption of gonad morphology in rainbow trout male juveniles chronically exposed to EE2 (0.01 to 10 µg/L), together with changes in gonad sex steroid levels and gonad and brain gene expression. A concentration dependency of the effects was evident: at lower concentrations of EE2, ovotestis gonads pre-dominated, while the gonads were sex converted at the higher concentrations. Intersexuality has been described using several terminology in the literature. Here, we refered to the term “ovotestis” to describe the occurrence of oocytes in a male gonad [47]. We use the more broad term “intersex” (occurrence of either female gametes in a male gonad or male gametes in a female gonad) to refer at the litterature. In gonochoristic species, intersex fish are fish with gonads displaying male and female characteristics simultaneously. They are observed at low prevalences in wild populations (*e.g.* in gudgeon [20], [64]), including in the obvious absence of estrogen exposure [2], [65]. The release of endocrine disruptive compounds into the environment has been shown to be an environmental inducer of intersex gonads through field and laboratory-based experiments [7], [21], [31]. The morphological appearence of intersex gonads appears to vary among fish species, but also with the type of steroid-active chemical and its concentration, the route and duration of exposure, and the timing of exposure (*i.e.* developmental stage of the fish) [65].

In rainbow trout, intersexuality has been rarely observed in the wild (only two cases of apparently natural intersex have been reported to date [66]), and there are no reports of intersex at contaminated sites in several trout species [27], [33]. Several studies investigating the effects of effluents or EDCs (including EE2) did not observe ovotestis [37], [67], and the sex ratios of exposed groups were unchanged compared with control groups. The few cases of disruption in gonad morphology of salmonids environmentally exposed to estrogens that have been reported in the literature include a high degree of vacuolation of the testis in brown trout (*Salmo trutta*) from a stream impacted by sewage effluent [34], and an intersex individual in brown trout exposed to a high dose of E2 (0.5 µg/L) at the juvenile stage [35]. For rainbow trout, inhibition of testicular growth was found after developmental exposure to estrogenic alkylphenols and EE2, but in this stduy gonad histology was not examined [27]. Consistently with the laboratory-based study of Krisfalusi in 2000 [67], our study showed that gonad morphology in rainbow trout is sensitive to feminization by environmental estrogens. However, it confirmed that high levels of xenoestrogen exposure are necessary in trout fry to change gonad morphology. It also appears that not only the concentration, but also the timing of exposure is of great importance in this process. Likewise, the results of Krisfalusi and Nagler (see below) [67]) suggest that trout may be more sensitive if exposed prior to the fry stage.

In the present work, the EE2 treatment resulted in different forms of ovotestis, ranging from single oocytes in otherwise normal tetsicular tissue (ovotestis 2), to testes with areas ressembling early ovaries (ovotestis 1) over mixed testicular-ovarian gonads (ovotestis 3) to fully reversed ovarian-type gonads. To our knowledge, the first feature observed (ovotestis 1) has never been reported in rainbow trout or other species submitted to xenobiotics. A high proportion of the ovotestis fish belonged to this category (51%). This feature is characterized by early ovigerous lamellae and actively dividing oogonia grouped into nests at the periphery of the testis. This peripheral structure looks like ovary tissue at the early differentiating stage (6–9 weeks of development) as described by Cousin (1988) [61], which could correspond to delayed female tissue, as the fish were 14 weeks old at the sampling time. The second ovotestis category observed (Ovotestis 2) here has been extensively reported in studies on the morphological impact of EDCs in several fish species (for an extensive review, see [65], Table 6.2). Ovotestis has been observed in rainbow trout following exposure to high doses of exogenous hormones [35]. Interestingly, Krisfalusi and Nagler (2000) [67] observed ovotestis in genetically male rainbow trout juveniles after only two 2-hour immersions 7 days apart in 250 µg E2/L. They observed up to 63% intersex fish and found that the labile period in rainbow trout is between 44 and 51 dpf. Our results support this greater sensitivity of rainbow trout to exogenous steroid treatment during the early sex-differentiating period. The last feature observed (ovotestis 3) is similar to asymmetric intersex described in the literature in several fish species exposed to EDC (*e.g.* Zebrafish [68], Whitefish [69]). Cases of asymmetric gonads were reported during attempts to achieve inversion in juvenile rainbow trout treated with high doses of estrogens (30–60–120 µg/g estrone) [70], and in a *mal*-mutated family of rainbow trout [66]. Such cases can also occur in feral fish populations, although the reasons for this are not known (*e.g.* [71]). The relative frequency of the different types of gonads varied with the EE2 concentrations, suggesting that this represents a series of events, from initial, slight disturbances in normal germ cell differentiation in response to the presence of elevated estrogen signals, to the full conversion of gonad physiology. This interpretation was corroborated by the gene expression results and sex steroid analyses (see below).

The EE2 treatment did not simply shift the gonads from a testicular to an ovarian morphology, but also induced certain alterations in testis structure (see results section 3.1.2). Pathological alterations have been reported in testes and ovaries after exposure to EDCs in several fish species (for an extensive review, see [65], Tables 6.3–6.12), and could be due to a toxic effect of the treatment. Considering that a 96-h LC50 of 1.6 mg/L was reported for EE2 in rainbow trout [72], the alterations observed at doses 160 thousand times lower (i.e. 0.01 µg/L) attest of the powerfulness of EE2 action *in vivo*. A deeper histopathological analysis of the testicular morphology is beyond the scope of this study, and will be addressed in future studies. Here, we will discussed the major pathological features observed, described in the results section 3.1.4.

The most intriguing pathological feature was the appearance of an epitheloid ciliated structure starting at the 0.1 µg/L concentration, which became more prominent with the increase in the EE2 doses. The “proliferative oviduct-like epithelioid” structure has never been reported in juvenile or mature rainbow trout females, either under natural or experimental (including toxic) conditions. The epitheloid designation is based on the fact that the tissue did not strictly correspond to the definition of an epithelium according to Grier *et al.* [63], as it did not border any body surface, lumen or duct and appeared to be vascularized. Moreover, Mac Millan [60] concluded that most epithelia of teleost oviducts are composed of ciliated cells interconnected by junctional complexes. However, salmonids display primitive ovarian features, the rudimentary ovarian cavity being continuous with the body cavity on its ventral side [46], [73]. They have no oviducts and the ova are discharged directly into the coelom [46]. The “proliferative” appellation refers to the fact that the structure increased in size with the increase in EE2 concentration. Several histopathologically oriented investigations of endocrine disruption have also inspected gonadal duct formation, and the results generally mention induction of this structure [7], [12], [74]). Our results are supported by the fact that estrogens play an essential role in stimulating growth of the female reproductive tract (including oviduct) [65]. Interestingly, Cousin [61] reported a high proliferation of the efferent duct ciliated epithelium in male rainbow trout juveniles exposed to 17α-methyltestosterone.

Another particular morphological feature in the gonads of EE2-exposed trout was the presence of mature follicles. Physiologically, rainbow trout develop mature follicles no earlier than after 2 years of age. Folliculogenesis (or oogenesis) is the complex process by which a diploid oogonia develops and matures into a haploid ova [60]. This process has been extensively described in fish, and the number of developmental stages described depends on the species studied and the authors [45], [46], [62], [75], [76]. In rainbow trout, follicle maturation has been divided into three main phases (previtellogenesis, exogenous vitellogenesis and maturation), with seven oocyte stages, depending on their histological features [62]. During this process, the oogonia undergo mitotic and meiotic divisions, become surrounded by follicular cells which specialize into granulosa and thecal cells, increase dramatically in size and accumulate yolk as the egg reserve during vitellogenesis. In mature fish, this process normally extends over nearly 8 months [25]. In juveniles, the ovary displays only stages 1 and 2 of previtellogenic oocytes [62], its size does not exceed 110 µm and it is surrounded by a thin follicule layer. The vitellogenic process only starts at the first maturation when fish are around 2 to 3 years old. In the present study, some isolated mid-vitellogenic oocytes were found in 4-month-old genetic males. This feature has never been reported thus far. Moreover, no intermediary follicular maturation stages were seen, and the mature follicles appeared isolated from the gonads. Thus, the action of EE2 seems to override oogenesis, maybe acting as a major actor in this maturation pathway. Whether these effects resulted from the action of EE2, or involved additional systemic processes implemented by the chronic treatment, cannot be determined from the data obtained in this study.

The expression of most of the genes tested was significantly regulated by the treatments and displayed a clear response pattern, with linear or optimal-response curved. In the testis, *cyp11b2.1* expression appeared strongly suppressed by the EE2 treatment. We also observed linear downregulation of *dmrt1* and s*ox9a2* with increasing EE2 concentration, which is in line with the change of gonad morphology from testis to ovary. These findings also confirm the status of *cyp11b2.1*, *dmrt1* and *sox9a2* as early testicular markers [77], [78]. However, unlike the conclusions of Vizziano in her study (2008 [78]), *dmrt1* response to EE2 was more marked than s*ox9a2. sdY* has been recently described as a master sex-determining gene in rainbow trout [26]. To our knowledge, this is the first time the expression of this gene is investigated under chronic exposure to exogenous estrogenic hormone. The significant decrease observed in EE2-treated fish highlights a down-regulation of its expression by exogenous estrogenic treatment, in a dose-dependant manner. This reinforces its role in testis differentiation. As this gene has been found to be mainly expressed during early testicular development, with a peak of expression between 60 and 90 dpf and a rapid falls thereafter, the relation between estradiol and *sdY* expression should be investigated further, at early developmental stage.

The specific ovarian differentiation marker *cyp19a1a* and the *esr1b* isoform displayed slight overexpression at low EE2 doses and significant underexpression at higher ones. This pattern was also observed for gonadal *vtg,* but with a sharp 90-fold increase at 0.1 µg/L and a marked drop at higher doses. The increase observed at low concentrations for the estrogen-responsive genes in gonads and brain may represent a positive response to the estrogen signal, but the subsequent decline could attest to a toxic effect of the higher doses of EE2 used. This is consistent with the decline observed in the size and weight of the fish and the pathological morphological features observed in sex-reversed fish at these concentrations. This pattern of expression (at least for *cyp19a1a*) may also reflect a negative feedback in response to the presence of high doses of exogenous estrogens.

Nuclear estrogen receptors (*ERs*) act as transcription factors mediating the expression of estrogen-dependent genes. Being not strictly specific, they are the first targets to trigger xenoestrogen action. Four ER isoforms have been described in rainbow trout (*ERα1–2; ERβ 1–2* encoded by *esr1a–b and esr2a–b* genes, respectively), with ubiquitous tissue distribution in juvenile fish, the highest ER mRNA levels being measured in the brain and testis [41]. Following xenoestrogen exposure, ER expression has been shown to increase in the gonads, brain and liver of several fish species [79]–[81], although in other fish species, no such induction has been described [82]. Our results are neither consistent with these results, nor with those obtained by Boyce Derricott (2009, 2010) who evaluated the expression of the entire ER family in rainbow trout juveniles exposed to a reversion dose of E2 and only found *esr1a* to respond, being upregulated in the liver and gonads [83], [84]. However, while the upregulation of ER mRNA in the liver by estrogens is characteristic of oviparous vertebrate systems (*i.e.* the increase in ER is necessary for VTG uptake during vitellogenesis) [85], the regulation of ER transcription in the gonads and brain is still poorly understood. Indeed, several studies have reported that the expression of different ER isoforms can differ widely within the same species and that xenoestrogens can show varying time- and dose-dependent patterns of activation, which also differ from that of E2 [86], [87]. Moreover, in rainbow trout, no sexual dimorphism is seen in the 4 ER expression patterns during gonad development [84]. Based on these considerations, we conclude that in rainbow trout fry, the responses of the different ER subtypes to estrogens vary between tissues, (*i.e.* the same receptor may respond differently if it is expressed in the gonads or brain) and most (but not all) ER isoforms respond to EE2 treatment in a dose-dependent manner. The ER response may vary as well with the developmental stage of the exposed fish, as different ER expression patterns have been observed in juvenile and mature fish exposed to exogenous estrogens [86].

The enzyme cytochrome p450 aromatase (*cyp19a1*), which converts androgens into estrogens, plays a key role in the control of ovarian and testicular differentiation. Its overexpression is needed to trigger and maintain ovarian development whereas its downregulation is necessary to induce testicular differentiation [88]. Several indications suggest that interference with the aromatase system could lead to malfunction of the reproductive system [89]. Two forms of this enzyme exist in fish, one mainly expressed in the gonads (*cyp19a1a*), and the other in the brain (*cyp19a1b*). Our results did not show any variation in gonad aromatase expression between control and EE2-treated fish. Only the brain aromatase expression showed an increase, at least at the highest concentrations. These observations would agree with the presence of ERE in the promoter region of *cyp19a1b*, while an ERE is lacking in the gonad form of aromatase [89]. Overall, exposure to xenoestrogens generally results in upregulation of *cyp19a1b* expression [90]–[93], whereas most studies conducted on fish feminization found no estrogen-inducible upregulation of *cyp19a1a* gene expression [78], [90], [94], with one exception in a study of EE2 induction of *cyp19a1a* mRNA in the testis of medaka [95]. Our results support the hypothesis that, in rainbow trout, feminization induced by exogenous estrogen treatment may not involve direct regulation by the aromatase pathway [88]. This is reinforced by the fact that neither E2 nor T levels significantly varied at all the concentrations tested in our study. On the other hand, the strong underexpression of *cyp11b2.1*, correlated with the decrease of 11-KT levels in EE2-exposed fish, draws attention to an important potential role of the 11β-hydroxylase in sex reversal in fish. This gene is expressed very early in differentiating gonads, with a sexual dimorphic pattern of expression visible from 45 dpf. Its expression has been also shown to be strongly and quickly suppressed by exogenous estrogenic treatment at very early developmental stage (from 53 dpf) [96]. Finally, although the initiation of ovarian development in female trout is mediated primarily by estradiol (E2), nuclear estrogen receptors (ERs), *cyp19a1a*, and, to an unknown extent, brain ERs and *cyp19a1b*, the lack of a correlation between the ER isoforms and *cyp19a1a–b* expression underscores the complexity of their relationship and the diversity of estrogen actions outside the sex differentiation process. Indeed, in the brain it has now been well established that estrogens play major regulatory roles in neurotrophic and neuroprotective pathways, besides their classical control of reproductive functions [97], . From this standpoint, the upregulation of *esr1a* observed in the brain, with no effects observed on *cyp19a1b* expression, could be related to these further functions of E2. Moreover, the gene expression results should be viewed with caution due to the involvement of those genes in transdifferentiation processes, as only one sampling point was analyzed, after a long exposure to the potent xenoestrogen EE2. Therefore, their expression pattern could reflect more complex responses involving other pathways and systems. Considering the sensitivity of rainbow trout to ovotestis induction at low doses of xenoestrogen, as highlighted in the present study, it would be interesting to study the mechanisms underlying this male-to-female transdifferentation processes in greater detail, focusing on low doses (below those used here) and very early developmental stages. Given that numerous genes should be implicated, and that the trout genome is being sequenced, it would be interesting to extend the study to the entire trout genome. It would also be of interest to study the reversibility of the morphological changes observed.

In conclusion, the fact that ovotestis has not been reported in field studies of rainbow trout does not mean that trout cannot develop ovotestis. The existing litterature highlights that the impact of estrogen exposure on gonad morphology, and therefore the propensity of a fish species to develop intersex/ovotestis, depend on a number of factors, such as the concentration of the xenoestrogen, the exposure duration and importantly the timing of exposure. The mode of sexual differentiation of a species, *e.g.* differentiated/undifferentiated gonochorist may be also of importance [99]. Interestingly, a study conducted by Lange et al. (2008) [100] on roach (*Rutilus rutilus*) showed that a single low EE2 concentration (4 ng/L) can lead to different effects depending on the exposure period/duration: a life-cycle exposure starting from fertilized eggs induced a 100% female population whereas exposure during the period of sexual differentiation induced intersex fish. Therefore both a certain estrogen level and exposure timing/duration are needed in roach to get an all-female population. From the results of our study it appears that trout is well able to develop ovotestis gonads, however, it needs exposure concentrations which are higher than what is usually found in the environment. Estrogens induce not only ovotestis but they can lead to complete testis-ovary conversion. The morphological changes are accompanied by corresponding changes in expression of testicular marker genes and sex steroid levels. No linear but rather complex relations were observed between ER/aromatase expression and estrogen exposure, indicating that these genes are not directly involved in the sex conversion process.
